# Global dataset of sand dam features and geographical distribution across drylands

**DOI:** 10.1038/s41597-025-06197-w

**Published:** 2025-12-08

**Authors:** Luigi Piemontese, Lorenzo Villani, Natalia Limones, Jeroen. C. J. H. Aerts, Giulio Castelli, Jessica A. Eisma, Bongani Mpofu, Doug Graber Neufeld, Hannah Ritchie, Cate Ryan, Ruth Quinn, Christine Whinney, Elena Bresci

**Affiliations:** 1 Department of Agriculture, Food, Environment and Forestry (DAGRI), University of Florence, Florence, Italy; 2 Department of Water and Climate (HYDR), Vrije Universiteit Brussels, Brussels, Belgium; 3 Department of Physical Geography and Regional Geographical Analysis, University of Seville, Seville, Spain; 4 Institute for Environmental Studies (IVM), VU University Amsterdam, Deltares Institute, Delft, Netherlands; 5 UNESCO Chair in Hydropolitics, University of Geneva, Geneva, Switzerland; 6 Institute for Environmental Sciences (ISE), University of Geneva, Geneva, Switzerland; 7 Department of Civil Engineering, University of Texas at Arlington, Arlington, Texas USA; 8 Dabane Water Workshops, Bulawayo, Zimbabwe; 9 Department of Biology, Eastern Mennonite University, Harrisonburg, Virginia USA; 10 School of Water, Energy and Environment, Cranfield University, Cranfield, UK; 11 Department of Environmental Science, Auckland University of Technology, Aotearoa, New Zealand; 12 Department of Civil Engineering and Construction Studies, Atlantic Technological University Sligo, Sligo, Ireland; 13 Sand Dams Worldwide, London, UK

**Keywords:** Water resources, Civil engineering

## Abstract

Sand dams are water infrastructure, built across ephemeral sandy rivers, that increase water supply by creating an artificial sandy aquifer upstream of the dam. Despite their effectiveness and recent traction in the research and development arena, empirical data on their distribution and characteristics are scattered and largely unreported. This gap represents a major barrier for understanding the large-scale potential of such a Nature-based Solution and for planning new installations. This paper presents a global dataset of sand dam locations and dimensions, developed collaboratively by research and development experts. We collected sand dam information from several sources, including local sand dam organizations. The data was reviewed and integrated through visual inspection in Google Earth. Although most georeferenced sand dams are from Eastern and Southern Africa, this dataset is a first global inventory and represents an invitation for others working in sand dams around the world to contribute their data. The dataset supports research on the effectiveness of sand dams and can aid practitioners with science-based criteria for sand dam development.

## Background and Summary

Small water infrastructure, such as ponds and small dams, are fundamental to support agricultural activities, especially in rangelands, where they often represent the only reliable water sourcE^1^. Sand dams are a particularly promising water infrastructure for drylands, since they provide specific benefits compared to conventional small dams^2^. In fact, sand dams are concrete walls built across ephemeral streams, which are specifically designed to trap sandy sediments upstream of the dam, thus creating an artificial aquifer able to increase shallow groundwater resources. The main elements of a sand dams are (i) the dam wall, which is anchored to the bedrock (or to a deep compacted clay layer) and raised for at most 2 m above the sand, (ii) spillways to allow runoff to flow downstream together with fines sediments and wings to anchor the structure to lateral rocks (or anchored deeper into the terrain). The water contained within the sand pores is protected from evaporation and, to some extent, from surface contamination, making sand dams a nature-based solution to water scarcity in drylands^3^. Although the first known sand dams date back to the early 1900s^4^, their construction is increasing exponentially over the last couple of decades thanks to the effort of several non-governmental organizations (NGOs) working on sustainable rural development.

It is helpful to differentiate and contextualize the sand dams within the broader family of in-channel water harvesting structures, which all harness seasonal flows to improve water availability in drylands. Among the most comparable are check dams, subsurface dams, and water spreading weirs, each with different strengths and limitations depending on local hydrology, terrain, resources and intended use.

Check dams are widely used for erosion control and some groundwater recharge. They are normally relatively simple and low-cost to construct, and due to their usual small sizes, they have an impact normally if several are constructed in the same area. They effectively reduce runoff speed, promote sediment deposition and landscape stabilization, and enhance some infiltration along the streambed and adjacent banks^5,6^. Unlike sand dams, they do not typically store significant volumes of accessible water for dry season use, as this is normally not its purpose.

Subsurface (or underground) dams, constructed beneath the riverbed, are highly effective at blocking the downstream flow of groundwater and storing water below the surface^7^. One of their advantages is structural flexibility—they do not require above-ground stability, making them suitable for wider channels and more downstream locations, so they normally have a larger storage capacity than sand dams, which in turn can potentially have more upstream and downstream impacts. Subsurface dams typically require more extensive excavation, and, unlike sand dams, they do not rely on sediment accumulation to form a surface reservoir. As a result, their ability to facilitate lateral connectivity with bank storage depends largely on the geomorphology of the channel and surrounding banks. Accessing water from subsurface dams generally requires deeper wells or more robust pumping systems, making them less accessible and more costly for community use.

Last, water spreading weirs are designed to slow floodwaters and spread them laterally across the floodplain, enhancing soil moisture, supporting vegetation regrowth and flood-based agriculture, and recharging shallow aquifers^8^. Their strength lies in improving agricultural potential and landscape productivity over a broad area. However, they do not store water directly within the channel and are not intended for abstraction.

A renewed interest in sand dam structures arose in academia, with several new studies and reviews addressing the many research gaps on sand dams’ hydrological and socio-economic aspects^2,9–11^.

However, both research and practice on sand dams are hindered by limited data availability on sand dams’ locations, characteristics, and impacts. The main reason for this data void is because most historical structures were not reported or monitored after they were built^12^, and recent sand dam development is driven by NGOs, which often do not systematically share their records. While large-scale datasets are often available for traditional dams of varying dimensions, including large and medium dams, both at the national and global scale^13–15^, no datasets have been produced for sand dams, despite representing a pivotal water infrastructure in drylands, where water is most needed. The reasons for this data gap are that i) the majority of sand dams are built by local communities, thus data are not systematically reported, ii) they traditionally have small dimensions, making their identification from satellite images difficult. In fact, most traditional dam datasets are derived from remote sensing of open water bodies, which is impossible for sand dams, since they do not store visible open water except for very short periods (often just a few days per year).

Here, we present an extensive and revised dataset with 1006 records of sand dams across the world, the Global Sand Dams Dataset (GSDD). We gathered data from different available sources and developed a network of researchers and practitioners to share, enrich and revise information on dams’ characteristics, such as dams’ crest length, throwback, and stream width. The scope of the dataset is to provide open access data to boost research and improve the understanding of sand dam suitability and impact, which can eventually support the implementation of new sand dam construction programs. The dataset can also serve as a resource for those interested in exploring various factors that influence the success of development projects, including questions from anthropology and other socially engaged research domains. As remote sensing data availability increases and artificial intelligence-supported analysis techniques expand, the dataset also offers an opportunity for researchers across the spectrum of earth, social, and agricultural sciences to conduct novel research into rural food and water security in drylands. Research-quality data from rural areas in the Global South remains rare or at the very least, inaccessible, and this dataset seeks to provide an example of how such data can be stored and shared, in accordance with the Findable, Accessible, Interoperable, and Reusable (FAIR) data principles. The launch of the dataset is also an open call to anyone interested in sharing further data on sand dams, providing a platform for systematically collecting and harmonising sand dams reporting standards to advance research, sharing knowledge and foster implementation and replication/learning from previous experiences.

## Methods

In this section, we i) present an overview of the dataset production procedure, ii) describe the data sources, iii) report the data harmonization and enrichment process, and present the GSDD attributes and features.

### Dataset production procedure

**Fig. 1:**
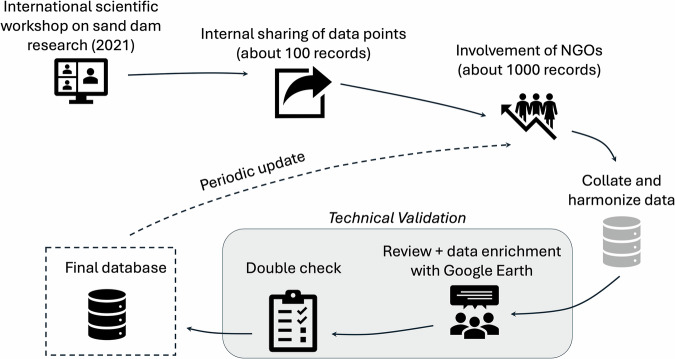
Steps involved in the production of the GSDD.

The collection, development, and technical validation process of the GSDD is concisely displayed in Fig. 1 and explained in detail in the sections below.

The working group started in May 2021 during an online workshop on sand dam research organised by the Water Harvesting Lab of the University of Florence, Italy (Castelli *et al*. 2022). The working group later expanded, including Sand Dams Worldwide and Dabane Trust Water Workshop, which are two major NGOs working on the construction of sand dams.

A first set of 110 records was collected by merging all data points provided by the working group of international researchers and collaborators based on sand dams they have personally worked on, either during the construction process or during research projects. Since most of those 110 records were shared by the NGOs involved in the funding and construction of sand dams, we opened the collaboration on the dataset to the major NGOs working with sand dams. At this stage we collected the majority of the records in the GSDD, which was later finalized with the integration of records from scientific and grey literature, and open sources online platform. Given the diverse data sources, we designed a multiple step technical validation procedure involving a careful screening and visual inspection of all records and a double check of a random sample of the dataset. These steps are described in detail in the following sections.

### Data sources

The data sources are provided in the attribute “Source” of the dataset. The aim of the GSDD is to make core information on sand dams’ location and characteristics available for researchers and practitioners to eventually improve their spread and effectiveness. Since most sand dams are built by NGOs, the main data sources are the NGOs involved in the funding, planning or construction, mostly in rural communities. The main international NGOs providing data are Sasol Foundation (https://www.sasolfoundation.com/) and Sand Dams Worldwide (https://www.sanddamsworldwide.org.uk/ – previously Africa Sand Dams Foundation), while Dabane Trust (https://dabane.org/) is located in Zimbabwe and No One Out in their local hub in Uganda (https://www.nooneout.org/). These NGOs account for most of the records in the GSDD, which they provided as csv with the available information based on the attribute of the GSDD that we shared with them as a template in advance.

The minimum requirement for the records to be included in the dataset is the geographic location coordinate. However, many records collected along the process have more details. For example, the initial set of 110 records shared by the researchers of the GSDD team also have information on construction date, presence, and type of water access (e.g. scoop holes or solar pumps etc.) or purpose of the sand dams (e.g. domestic use or pastoral use etc.). Additionally, the 110 records have additional information on water quality, because of the analysis conducted during a collaboration between the NGO Africa Sand Dam Foundation and researchers from the GSDD team. Although none of the other data collected from other sources have such information on water quality, we kept those attributes as a blueprint for future research.

We also included the very little freely available data on sand dams from online open access sources, including two records in the Global Database on Sustainable Land Management (WOCAT^16^, located in Kenya and three records from the Global Inventory of Managed Aquifer Recharge applications by the International Groundwater Resources Assessment Centre (https://un-igrac.org/our-work/activities/global-inventory-of-managed-aquifer-recharge-schemes/). From these sources we only kept the location and the construction date, before enriching them with the other attributes described in the next sections.

From scientific and grey literature there is mention of sand dams in several countries, including Namibia, India, and South Korea^4,10^, but locations remain mostly unknown. From these sources, we created records of the sand dams with known locations and cited the source in the “source” attribute either as a scientific reference or as a website. Finally, we have checked the Global Dam Watch (GDW) database^17^ (i.e. the most complete dataset of river barriers and reservoirs) for sand dam structures. Since we found no overlap with our GSDD, we can reasonably deduce that the GDW does not include sand dams records.

### Dataset harmonization

Because of the diversity of data sources and reporting standards of the preliminary dataset, a thorough harmonization process was designed and implemented to 1) avoid redundancy in the records, 2) provide a coherent structure, aligning all records and setting common attributes, and 3) check and adjust the reported location (LAT and LON coordinates) of the dams. Since the whole compilation (and review) was based on visual inspection on Google Earth, thus subject to the researchers’ judgement, we organized internal online workshops among the core team of co-authors involved in database compilation and review to ensure consistency across records. During these interactions we collectively defined a set of shared criteria and steps for verifying and enriching records. For example, we agreed on consistent visual inspection procedures using Google Earth to confirm the presence and characteristics of each sand dam. We also established specific guidelines on how to measure dam crest length, throwback distance, and stream width — ensuring that all researchers interpreted and measured these attributes in a uniform way (like throwback measured as straight-line distance upstream from the dam, width measured at regular intervals along the throwback).

Specifically, if the sand dam could not be located on Google Earth, the reason was provided as:

- Not visible from Google Earth – either if the sand dam could not be found or if the quality of the satellite image was not sufficient (e.g. cloud coverage or very low resolution).
- No image available on Google Earth – the sand dam was built recently, and no satellite image was available.
- Off-stream location – the coordinates were located off a waterway, and presumed to be inaccurate, since no dams were found in the surrounding area.
- Broken – the sand dam was reported as broken or the dam is visibly broken from the satellite inspection, either collapsed or partly damaged.
- Water pond – the coordinate point to a structure which holds water all year round.

4. If additional sand dams were identified during the process, they were added in the dataset.

5. Add comments about the sand dam, if needed.

6. Enrich the dataset with additional information, including the following dam and stream characteristics, which are key for estimating the sand and water storage potential:

- The length of the dam structure, including the dam’s main doby and the visible portion of the wings
- The throwback, which is the longest straight line from the dam’s body upstream.
- The average width of the river upstream of the dams along the throwback.

The dam and stream characteristics were retrieved by visual inspection from Google Earth. Although there are other high resolution satellite products, we use Google Earth, since it is the highest resolution tool among the freely available satellite tools, and widely used in peer-reviewed scientific dataset^14,18,19^. In fact, it allows for a transparent methodology and easy replicability or check from third parties. Additionally, the “time machine function” on Google Earth was instrumental for estimating the potential construction date of sand dams (i.e. picking the date of the image when the dam is first visible), when this information was missing from the original source.

## Data Records

Details of the attributes included in the GSDD^20^ are reported in Supplementary Table 1. As the core information of the GSDD are the location and characteristics of the sand dams, the geographic coordinates (i.e. LAT and LON), dam length, throwback and river width are the only complete attributes for all the records. We added a “comment” column in which additional information on data quality or uncertainty in the evaluation of sand dams’ characteristics are reported. For transparency, we also maintained comments related to the review process. The other attributes report information retrieved from the original sources; hence they are available for a subset of records. Values in the “construction date” column mostly consider the original source, except those estimated using Google Earth.

**Fig. 2:**
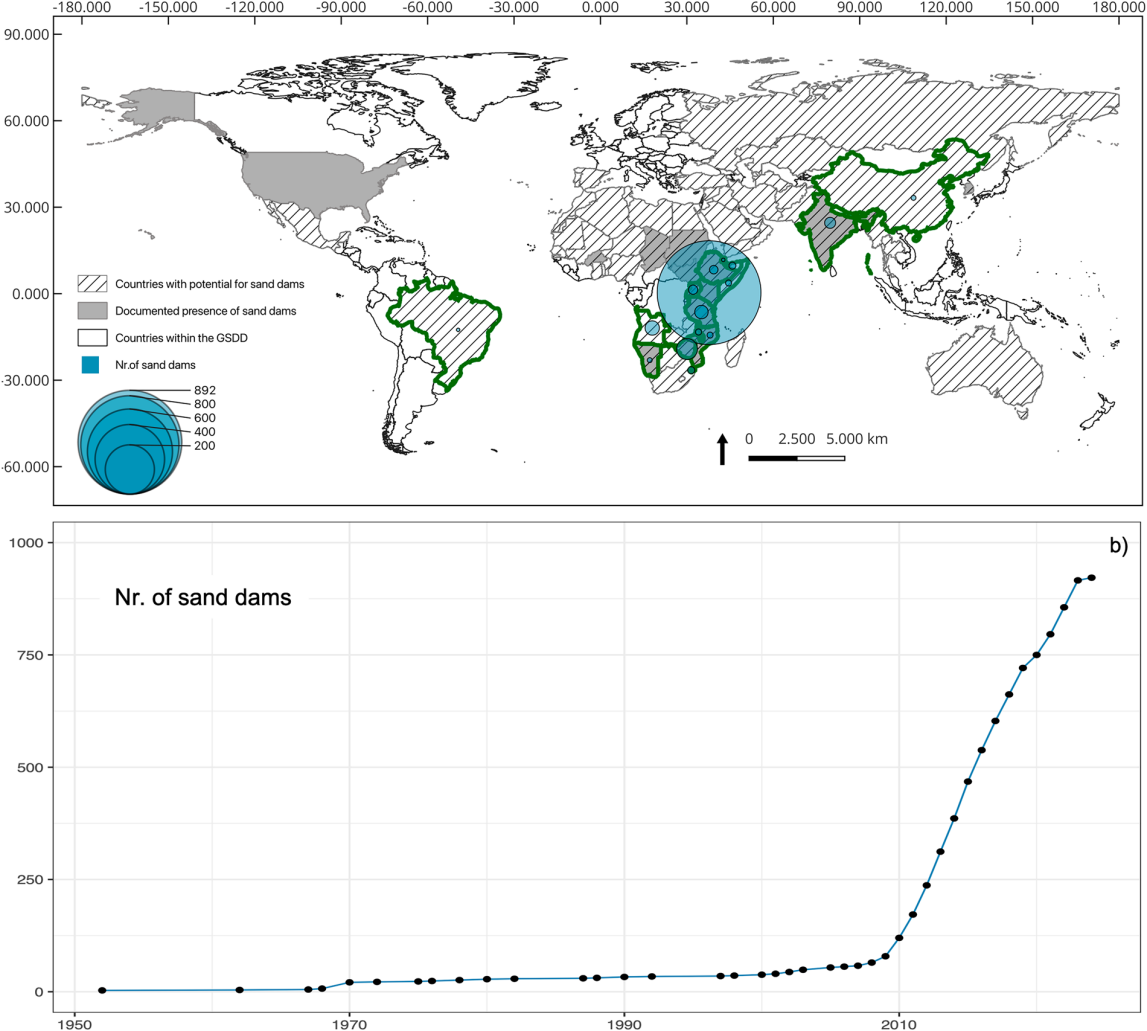
Spatial global distribution of the sand dam records of the GSDD, compared to countries with known presence of sand dams and potential countries suitable for sand dams according to (Yifru *et al*.^10^) (**a**) and the temporal trend of sand dam presence in the period 1952–2023 (**b**).

The dataset includes 1006 records, spanning across 15 countries and 3 continents (Africa, Asia, and South America), which were built over the last 80 years (Fig. 2). Most of the dams are in Kenya (892), including some of the oldest records from 1952. Although this seems to portray a skewed representation of the global spread of sand dams, it is in line with current scientific literature, which show that most sand dams are actually located in Kenya. For example, Ritchie *et al*., (2021) states that “the overwhelming majority of sand dams have been built in South-eastern Kenya, perhaps due to unique and favourable physiographic features, and thus the majority of sand dam research has also been conducted in Kenya”. Our dataset widens the range of countries hosting sand dams by including records from literature and research outside Kenya to provide a more complete picture of sand dams global spread and potential. Table 1, in fact, shows that Angola, India, Tanzania and Zimbabwe have over 10 sand dams in their territory.

**Table 1:** Country statistics of total number, mean and standard deviation (sd) values of sand dam characteristics.

Country	Nr. dams	Dam crest length (m)		Stream width (m)		Throwback (m)	
		*Mean*	*Sd*	*Mean*	*Sd*	*Mean*	*Sd*
Angola	17	44.7	33.3	37.1	37.8	282.9	230
Brasil	1	35	—	60	—	151	—
China	2	125	35.4	140	14.1	2250	1060.7
Djibouti	1	38	—	40	—	550	—
Eswatini	4	43.7	16.6	17	.4.2	227.3	97
Ethiopia	6	35.2	10.7	20.5	10.6	179.5	68.7
India	10	36.1	20.7	22.5	23.6	155.6	72.6
Kenya	892	35.7	16.3	19.5	10.8	162.5	14.4
Malawi	5	24.5	2.1	20.5	7.8	85	41
Mozambique	3	47.7	4.0	19.3	9.3	123	20.9
Namibia	2	51.5	2.3	50	28.3	655	657.6
Somalia	7	93.7	56.8	75	35.4	453.8	271.3
Tanzania	13	25.3	10.6	15	4.7	211.7	113.8
Uganda	7	53	9.5	32	14	264.7	30.7
Zimbabwe	36	56.2	23.2	22	10.8	278.2	170.5

**Fig. 3:**
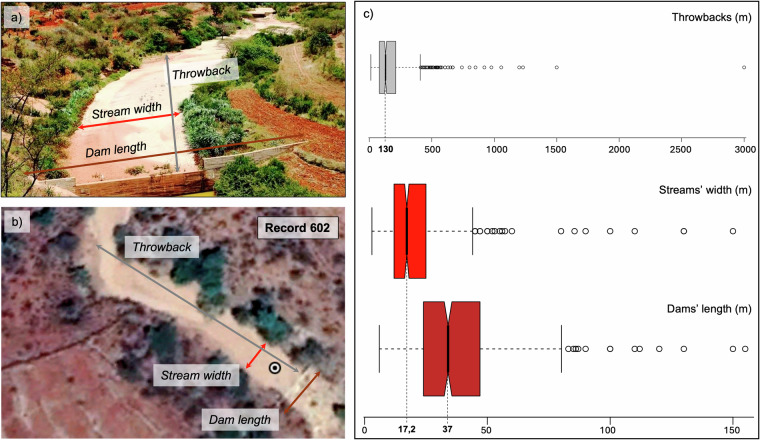
Median dams’ characteristics, including throwbacks, stream width, and dam dimensions (**a**), photo of a sand dam shared by Sand Dams Worldwide and its characteristics (**b**) and a snapshot with dam’s characteristics from Google Earth (**c**).

The median dimensions of the dams in the GSDD are 32 m of dam length, 17 m of stream width (upstream of the dams) and 130 m of throwback, although some sand dams show greater dimensions (Fig. 3). For example, the two sand dams in China are about 150 m in length, built across a stream of about the same width and quite straight, with about 3 km of throwback. The median country statistics are reported in Table 1.

## Technical Validation

A core team of 10 co-authors had volunteered to perform the technical validation of the dataset. The total records were split equally between the 10 reviewers, who checked all records individually on Google Earth.

Although the review process was very detailed and carefully coordinated with the internal workshops, it was still subject to the reviewers’ judgement. To limit uncertainty in records evaluation, a second review round was conducted on a subset of data including i) a set of records judged particularly unclear by the reviewers during the first review round and ii) a randomly selected subset of 200 records (accounting for about 20% of all record), for a total of 263 records. The second review round followed the same procedure as the first review except that it was conducted by different researchers. On average, about 80% of the records were confirmed in the second round, showing a satisfactory homogeneity in the imagery interpretation.

### Dataset limitations

This dataset is just a first step towards providing a complete picture of the global distribution of sand dams, although several challenges might hinder a comprehensive assessment. In fact, many sand dam projects, especially those initiated by farmers, local communities, or small NGOs, often lack formal monitoring and evaluation mechanisms, resulting in their exclusion from broader development databases and scientific literature. Many local sand dam projects may not have digital records or may be documented in formats that are not easily accessible or shareable.

These local or small-scale initiatives may not attract media or scientific attention, particularly if they are in remote or under-represented areas. Documentation and formal reporting efforts typically focus on larger, high-profile projects funded by international organisations or governments, overshadowing the achievements of smaller, community-led endeavours. Linked to this, efforts to gather data may be concentrated in regions with higher visibility or funding incentives for these topics. Fragmented data systems at local, national, and international levels further contribute to the invisibility of these projects.

Additionally, language barriers may impede information sharing about sand dams, with documentation often existing in local languages that do not reach the global community. Moreover, the terminology used to describe sand dams can vary across regions and organisations. Terms like “sand dam”, “subsurface dam”, “sand storage dam”, “ground-water dams”^21^ or even local names in regional languages may be used interchangeably to refer to similar structures, or these might be even referring to different structures than those inventoried in this work. Depending on the geographical region, there may be localised terms or colloquial expressions used to describe sand dams. An example is the term “barragens”, which in Angolan Portuguese refers to both conventional dams and sand dams interchangeably, while the local term “Chimpaca” is mostly used by Angolan pastoralists to refer to sand dams or cattle water ponds^9^. The language issue might also fuel misunderstanding between different water infrastructure and their spread. For example, of the many small dams in India documented by Yifru *et al*.^10^, the vast majority seem to be check dams or subsurface dams, as mentioned by^22^, but sometimes included in the geographical domain of sand dams. Moreover, even differences in approaches for construction can lead to labelling the infrastructures as different techniques, making it difficult to standardise data collection and reporting. The researchers used a variety of multilingual keywords and search terms relevant to sand dams in different languages, but the diversity of regional languages and dialects may still result in incomplete data capture.

This paper is an initiative to highlight the importance of including local projects that often go unrecognised. The authors aim to motivate others to also incorporate these smaller or community-led interventions into the dataset. Such inclusion and updates are necessary to render the dataset adaptable, transcending its status as a static depiction of sand dam locations.

Beyond the challenge of compiling cases, the dataset does not include *in-situ* measurements to refine the assessment of potential for water harvesting, nor performance metrics to understand if the sand dams are properly functioning (e.g. storing sandy sediments) or fulfilling their intended purposes.

### Potential Use and future development of the dataset

Notwithstanding the mentioned limitations, the GSDD offers a first usable tool to support researchers and practitioners in several endeavours. Researchers can use the GSDD to explore numerous research questions, which are at the forefront of hydrology research in water infrastructures. Some potential macro-research questions include (i) exploring the hydrological, ecological or socio-economic impacts of multiple sand dams at the catchment scale, including effects of upstream-downstream connectivity and water availability, (ii) assessing the effect of sand dams location and development on land use/cover change and social-ecological dynamics of agro-pastoralists, (iii) estimating the potential for water storage increase, or groundwater recharge, and its potential use, (iv) improving or validating large-scale best siting approaches and (v) evaluating socio-economic impacts and potential of sand dams implementations. The mentioned potential research frontiers are in line with recent collaborative scientific work.

While the research advances enabled by GSDD can support practitioners in improving sand dam implementation and effectiveness, the dataset can also be used as an operational tool, for (i) exploring new areas for sand dam projects based on geographical similarity and gaps, (ii) assessing the overall impacts of past and current sand dam projects and (iii) estimating dimensional characteristics of construction and its costs.

Although the majority of records currently come from Kenya, the dataset is not restricted in scope to this context. In recent years, sand dams have been increasingly promoted and implemented beyond Kenya, driven by international NGOs such as Sand Dams Worldwide and other global actors. Projects have been piloted or expanded in diverse socio-ecological settings, including South Korea^23^, Zimbabwe^24^ and Mexico^25^, among others. Some of these emerging structures are not yet systematically mapped, but their presence illustrates the rapid and ongoing spread of the technology. The very motivation behind the GSDD is therefore to provide a transparent, flexible, and extensible evidence base that not only documents the Kenyan experience but also accommodates and supports the integration of new data from other regions as it becomes available. In this sense, while Kenya dominates the current dataset, the GSDD contributes to the global research and development of sand dams, enabling comparative analysis, supporting scaling out in different contexts, and fostering co-learning for their sustainable implementation.

To provide further information in support of the mentioned objectives and beyond, a useful improvement of the database in a future publication could include a variety of hydroclimatic and socio-economic data for each data point, taken from local or global sources. For the hydroclimatic indicators, global data from TerraClimate, for example, can be used to extract variables such as runoff, precipitation minus evapotranspiration or climatic water deficit, helping assess water availability and the potential for water storage across different regions.

On the socioeconomic side, integrating globally available data can support characterizing community vulnerability in the environments in which dams are built, and potential impact of them in provision of water security. For example, population density from WorldPop could help estimate the number of actual or potential beneficiaries. The Multidimensional Poverty Index or livelihood information from UNHCR, for instance, could be integrated to provide insight into economic vulnerability. Data on access to improved water sources – for example coming from the WHO/UNICEF – can help contextualize the role of sand dams in improving water access and supporting climate-sensitive livelihoods.

Finally, the GSDD has the ambition to provide researchers and practitioners with a platform to improve data collection and monitoring of sand dams by expanding the network of contributors and users of the dataset. The ambition of the GSDD working group is to welcome NGOs and researchers in sharing data and experience on sand dam projects, contributing to improving the dataset’s comprehensiveness and quality with time, while supporting its use in research and implementation projects.

## Data Availability

The GSDD is an open-source platform, which can be expanded by integrating data from other global areas. The dataset is available on Zenodo, and it can be downloaded using the following URL: https://zenodo.org/records/15828863.

## Code availability

The data of the GSDD were processed and mapped using the software RStudio and QGIS. The R code is publicly available here: https://github.com/piemonteseluigi/Code_GSDD/tree/78147b27f26b08b6854c47e829f244e9abc2bdb0.
